# An Investigation into Picosecond Laser Micro-Trepanning of Alumina Ceramics Employing a Semi-Water-Immersed Scheme

**DOI:** 10.3390/ma12111812

**Published:** 2019-06-04

**Authors:** Qiang Ma, Hao Zhu, Zhaoyang Zhang, Kun Xu, Xueren Dai, Shuaijie Zhu, Anbin Wang

**Affiliations:** 1 School of Electric Power, North China University of Water Resources and Electric Power, Zhengzhou 450046, China; mqiang1977@ncwu.edu.cn; 2 School of Mechanical Engineering, Jiangsu University, Jiangsu 212013, China; zzhaoyang@126.com (Z.Z.); xukun@ujs.edu.cn (K.X.); dxueren@126.com (X.D.); shuaijiezhu@163.com (S.Z.); 17364399730@163.com (A.W.)

**Keywords:** Picosecond laser, alumina ceramics, semi-water-immersed, micro-trepanning, through hole formation mechanism

## Abstract

Intense interest has been given to the fabrication of micro-through-holes with smaller tapering and higher aspect ratios in engineering ceramics due to their wide range of applications in MEMS and aerospace. A semi-water-immersed laser micro-trepanning (SWILT) scheme is proposed and investigated in this paper with alumina ceramics as the target material, and its performance is assessed and compared with the direct laser trepanning method. Relevant processing parameters influencing the trepanning process are explored through an orthogonally designed experiment, and their effects on hole profiles are adequately discussed to yield optimized parameters. It is revealed that SWILT is capable of producing micro-through-holes with minimized hole tapering and much straighter sidewalls compared with the direct trepanning results, whereas the ablated surface quality is relatively rougher. The micro-through-hole formation mechanisms are also amply analyzed, where the transition hole development may be purely attributed to the laser-material interaction in the direct laser trepanning condition, while the SWILT case features an enhanced material-removal rate, especially at the lower part of the through-hole. The latter is due to the strengthened mechanical effects coming from the water-confined plasma zone and the following cavitation bubble collapse, which may efficiently expel the molten material from sidewalls and result in significantly reduced hole tapering.

## 1. Introduction

Engineering ceramics, typified by alumina (Al_2_O_3_) ceramics, is getting more popular in many fields due to its superior properties including high electrical insulation, high hardness and strength, high resistance to temperature, corrosion and wear and a low thermal expansion coefficient [1,2]. However, as a hard, brittle material, there exists a machinability issue in the micro-drilling of alumina ceramics using conventional methods, in which material cracks, tool wear and low efficiency often occur. Chang and Lin [3] used a peck-drilling scheme with a small feed rate and continuous cooling to overcome the poor machinability of alumina ceramics, where the tool wear was still inevitable during processing. In efforts to facilitate the micro-drilling of alumina ceramics using non-traditional methods, Wakuda et al. [4] conducted abrasive jet machining of alumina ceramics and explored the effects of abrasive particle kinds on the machined dimples on the ceramic surface. It was concluded that soft abrasives such as silicon carbide (GC) are associated with smooth surfaces but low material-removal rate, whereas hard particles such as synthetic diamonds (SDs) enhance machining efficiency, but also roughen the treated surface. Makino et al. [5] carried out a micropatterning study of alumina ceramics by the technique of photolithography, in which phosphoric acid (PA) was adopted to etch the material. However, the low material-removal rate may limit the application of this method, as the etch rate of alumina ceramics was only about 1 µm/min at temperatures over 250 °C. Similar suffering of low material-removal rates has also been found in ultrasonic drilling of alumina ceramics [6].

By contrast, laser may supply an efficient method of micro-drilling on alumina ceramics, owning to its advantage of focusing the pulsed energy into a small area to achieve high power density and cause material removal through melting, evaporating and exploding. Kacar et al. [7] reported that through-holes of diameters between 500 and 1000 µm can be machined in alumina ceramic plates of thicknesses around 10 mm using a microsecond (ms) pulsed laser, although dross and re-solidification regions of ejected material around the hole entrance were obvious. Similar drilling results can also be found in the ms laser drilling study of alumina ceramics carried out by Hanon et al. [8]. Nedialkov et al. [9] compared the ablation efficiency of alumina ceramics using nanosecond (ns) lasers of different wavelengths (i.e., 355, 532 and 1064 nm), and revealed that the maximum material-removal rate was achieved by the infrared laser, while the microcracks were clearly shown in the re-solidification region around the periphery under SEM observation. Bharatish et al. [10] examined the effects of processing parameters on the heat affected zone (HAZ) and hole circularity in the ns pulsed laser drilling of alumina ceramics. According to their study, entrance circularity showed an increasing trend with the laser power, but decreased with an increment in hole diameter, while larger hole diameters or laser power led to increased exit circularity. In addition, HAZ thickness appeared to decrease under a constant laser power when the pulse frequency increased.

Although there are reports that the micro-hole arrays on alumina ceramics can be drilled without cracking using high-density (around several GW/cm^2^) ns lasers under the appropriate process optimization [11], laser-induced damage including cracks, recast layers, dross and HAZ usually occurs in conventional laser (continuous laser or pulsed laser with duration from ms tons) drilling processes [10]. This is a result of the thermal material-removal mechanism in nature [12,13], which needs to be minimized as such defects may affect the functionality and reliability of the final products, especially for micro-machining.

To minimize the laser-induced thermal defects in drilling results, water has been introduced into the laser machining process by many researchers. Yan et al. [14] studied the underwater machining of alumina using a continuous CO_2_ laser, and found that the introduced water effectively helped reduce substrate defects such as cracks, dross, recast layers and HAZ that typically exist when machining in air. In addition, water layer thickness affects the machined kerf width significantly, and thus should be well prepared before machining. Barnes et al. [2] took advantage of the controlled thermal shock fracture mechanism for a machining purpose, in which the employed coaxial air stream generated a dry area on the alumina specimen for the laser to heat. This high temperature zone was then immediately quenched by the surrounding water as the laser spot swept away, leading to an improved cutting speed together with a tighter tolerance and good surface integrity. However, the laser beam and assisted water are mutually exclusive during machining at the given location, and therefore may not be suitable for the drilling action.

Reducing the laser pulse width to the ultrashort level (i.e., from 10^−12^ to 10^−15^ s) can effectively eliminate or minimize laser-induced thermal damage and improve the machined feature quality [15,16]. In a systematic investigation of the femtosecond (fs) laser-drilling of alumina ceramics performed by Wang et al. [17], high quality through micro-holes were achieved without recast layer, cracks and delamination when the processing parameters were properly chosen, confirming the feasibility of fs lasers in alumina ceramics micro-machining. Li et al. [18] conducted fs laser drilling of alumina wafers, and found that micro-holes of clean edges without cracking can be fabricated, and that particulates around the periphery of the hole caused by re-solidification of molten material were absent. It was also pointed out that a re-solidified material layer about 30 nm in thickness existed covering the wall, whereas no micro-cracks or modified micro-structure was observed within it. Ihlemann et al. [19] stated that the material-removal mechanism was switched from “plasma-mediated ablation” for ns pulse to multi-photon absorption, enabling material removal for an fs laser. As a result, µm-resolution features were structured by an fs laser without thermal influence to the surrounding material.

Since ns laser micro-drilling is usually associated with obvious thermal damage, while an fs laser is limited by high photon cost and the alleged low material-removal efficiency [20,21], there exists a trade-off between machining quality and efficiency [22]. Picosecond (ps) laser may be capable of achieving relatively high material-removal efficiency and good surface finish [16,23], offering a proper alternative choice in the micro-drilling of alumina ceramics. Hsu and Wu [22] carried out micro-via drilling on alumina ceramics of 380 µm in thickness using a ps pulsed laser, demonstrating that a through-hole of 72 µm at the entrance and 61 µm at the exit could be drilled efficiently with generally good geometric properties and a significantly reduced re-solidification region surrounding the periphery compared with ns laser drilling results, although debris deposition still can be observed on the wall inside the through-hole. Parry et al. [23] confirmed that ps lasers result in superior surfaces without cracking compared with ns laser machining, and such quality improvement can be translated to higher component strengths. More relative research about ps laser micro-drilling on alumina ceramics is missing, especially in terms of the potential exploration of aspect ratio improvement and mechanism analysis relating to the micro-hole formation process.

In this study, the ultrashort ps laser trepanning of alumina ceramics that is semi-water-immersed is conducted, and the influences of selected parameters on the trepanning performance are studied experimentally while employing an orthogonal design scheme. The machining results are evaluated under scanning electron microscope (SEM) observation and compared with the corresponding direct machining results qualitatively and quantitatively in terms of ablated surface quality and through-hole profile (i.e., entrance/exit diameters and hole tapering). Recommended parameters are obtained, and the optimized through-hole is therefore produced. Finally, the micro-through-hole formation mechanisms involved in the direct laser trepanning and SWILT processes are adequately analyzed.

## 2. Materials and Methods

### 2.1. Experimental Set-Up

**Figure 1:**
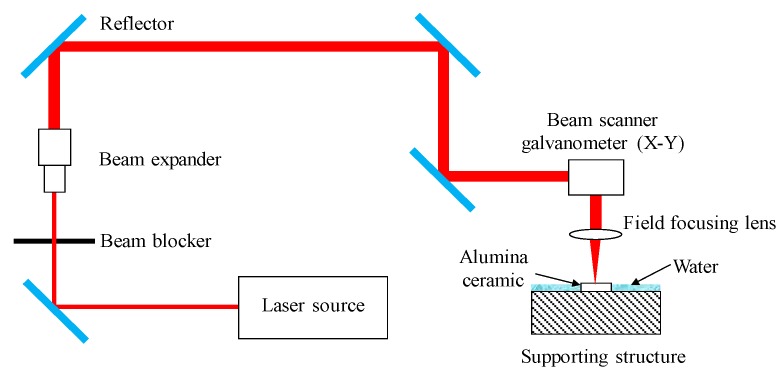
The schematic diagram of the ps pulsed laser trepanning system.

Figure 1 depicts the optical schematic of the laser trepanning system (Delphi Laser Co., Ltd., Suzhou, China), and a Nd:YVO_4_ laser (Edgewave PX100-1-GM, EdgeWave GmbH, Aachen, Germany) running in Gaussian mode was employed. The emitted laser beam propagates with a 12 ps pulse duration at a 1064 nm wavelength, while the output power theoretically peaks at 70 W. The laser pulse frequency can be adjusted between 0.2 and 1 MHz, yielding a maximum single pulse energy of about 260 μJ at 0.2 MHz pulse frequency, while the energy per single pulse decreases rapidly with an increment in pulse frequency. For this ps pulsed laser generator, the high voltage (HV) level operating on the HV modulator was used to modify the output power, as this power adjustment scheme is superior to regulating the pump current since the varied HV level has no influence on the laser spot size. In addition, a beam expander (Eoptics VE-532-1064, JENOPTIK, Jena, Germany) was included in the optical path to expand the emitted laser beam, then the enlarged beam was guided to a galvanometer (IntelliSCAN 14-1064, SCANLAB, Munchen, Germany) with a typical scanning velocity of 2 m/s and a focal length of 100 mm. Finally, the laser beam passed through the field focusing lens (TSL-1064-50-100Q, Wavelength Optoelectronics, Nanjing, China) before reaching the alumina ceramic surface to induce a laser–material interaction. For a safety protection purpose, a metal beam blocker was added in the optical system. To ensure the laser machine operated stably, filtered and deionized water was looped inside it to keep a constant temperature. Using this optical set-up, a focused laser spot with a diameter around 20 μm could be created in the focal plane.

**Figure 2:**
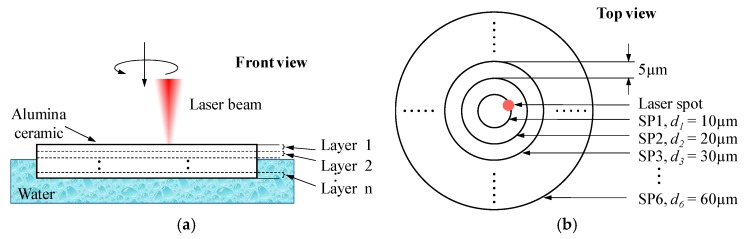
The schematic diagram of SWILT, where (**a**) and (**b**) represent the front view and top view, respectively. SP: scanning path.

In this experimental study, the target workpieces were rectangular alumina ceramic (95% of Al_2_O_3_, TO-247, Karefonte, Shenzhen, China) plates with dimensions of 25, 20 and about 0.6 mm in length, width and thickness, respectively. The main mechanical and physical properties of alumina ceramics are given in Table 1, while the main components and corresponding ratios are listed in Table 2. The target Al_2_O_3_ ceramic plates were produced through a hot-pressed sintering process, so a porosity structure may be expected in the following observation step. The target through-holes with diameters larger than the laser spot size were fabricated using a trepanning scheme, as shown in Figure 2, in which the laser beam feeds downwards according to the given layer number, circling the focused laser spot for each layer along six concentric scanning paths (SPs) corresponding to diameters of 10, 20, 30, 40, 50 and 60 μm, respectively. In this way, the final formation of the through-hole is created by the accumulated material removal corresponding to each laser scanning path at each layer.

In the SWILT process, a laser is employed to irradiate the ceramic plate and may result in melting, vaporization, plasma formation or direct phase explosion, as in fs laser machining case [16]. Consequently, material removal may be attributed to the explosion of vaporized or molten material, or the thermal-stress-caused fracture on the brittle ceramics. Once the ceramic plate is trepanned through, water will fill the micro-holes due to a siphoning effect, followed by laser-induced breakdown in the tiny space-confined liquid. This will result in a mechanical action on the sidewall via plasma expansion, cavitation bubble collapse and shockwave propagation, and thus more material removal is expected, especially at the lower part of the hole where the plasma and bubbles are more tightly confined (hence the reduced hole tapering). In the SWILT process, the horizontal alumina plate is partially immersed in water where the plate bottom is surrounded by water while the top surface is in direct contact with the air, as shown in Figure 2a. As long as the specimen bottom touches the water, the exact immersion depth of the alumina plate is not important, since water can be efficiently drawn up to the top plate surface via the micro-hole due to the siphoning effect.

### 2.2. Experiment Design

In this study, factors that affect the heating process may influence the material removal and, as a result, the micro-through-hole formation. To assess the trepanning process, the following parameters were chosen: laser power intensity (*I*), estimated as the ratio between the output power arriving at the specimen surface and the area of laser spot; laser repetition frequency (*f*); laser beam scanning velocity (*v*); element number (*m*), representing the repeated scanning time following a given scanning path; and layer number (*n*), corresponding to the feeding times of the laser focal plane position in a specific depth direction. The actual spot diameter was estimated to be around 33 μm using the hole burning method. The testing levels of each parameter were selected considering the preliminary test results and the available adjustment range in the laser drilling system, as given in Table 3. Four levels were selected for each processing parameter, and the experimental test was kept at a manageable size by using the orthogonal design scheme. Employing the L_16_(4^5^) orthogonal array with 16 rows and 5 columns, 16 sets of parameter combinations were obtained both for dry trepanning and for SWILT. In addition, to minimize the uncontrollable influence, each parameter combination was repeated three times, resulting in a total of 96 holes.

**Figure 3:**
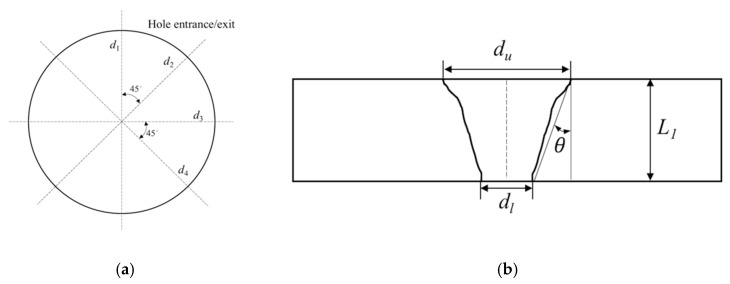
(**a**) Repeated hole diameter measurement method at entrances and exits, and (**b**) estimation scheme of the hole tapering [24].

Hole diameter measurements at exit and entrance sides, as well as the characteristic observations of the ablated surface were conducted after trepanning to evaluate the machining performance under different parametric combinations. The CCD microscope (IMAGING SOURCE, Bremen, Germany), which was combined in the advanced ps pulsed laser drilling system, was used to measure the hole diameters. For each hole, diameter measurements were repeated at four fixed orientations with an interval of 45°, as illustrate in Figure 3a. Consequently, each parameter combination led to 12 readings both at exits and entrances, and the average was taken as the diameter reading. The hole tapering was derived as

(1) $$\theta \;=\;\arctan\left(\left(d_{u}\;-\;d_{l}\right)/2L\right)$$

where *θ*, *L*, *d_u_* and *d_l_* correspond to hole tapering, ceramic plate thickness and averaged diameter of entrance and exit, respectively. In addition, the characteristics of the ablated surface surrounding the hole edge were also observed under SEM (Hitachi S-3400N, Hitachi, Tokyo, Japan and JEOL JSM-7800F, JEOL, Tokyo, Japan) for qualitative analysis. Moreover, to clearly study the effect of the trepanning process on the sidewall quality, selected through-holes were carefully ground and polished to obtain a section plane along the hole axis, then observed under SEM to help assess the trepanning result.

## 3. Results

### 3.1. Comparison between Direct Laser Trepanning and SWILT

**Figure 4:**
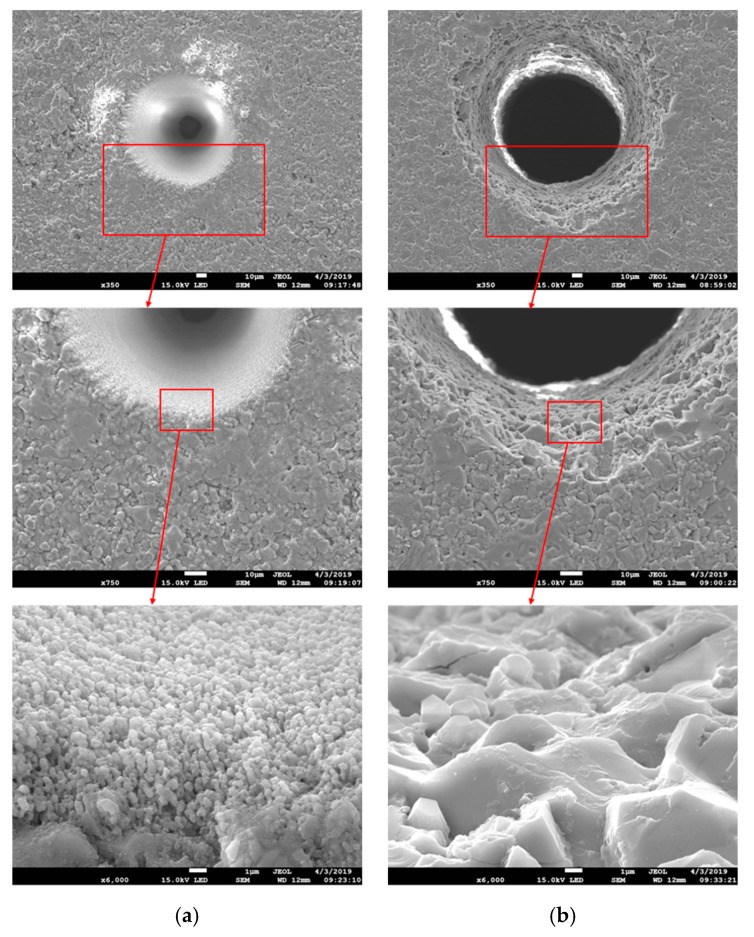
Comparison of the micro-through-hole entrances, in which (**a**) represents the direct laser trepanning result, while (**b**) corresponds to SWILT. The employed parameters for trepanning are: *f* = 260 kHz, *I* = 2.58 MW/cm^2^, *v* = 100 mm/s, *m* = 300, *n* = 20.

**Figure 5:**
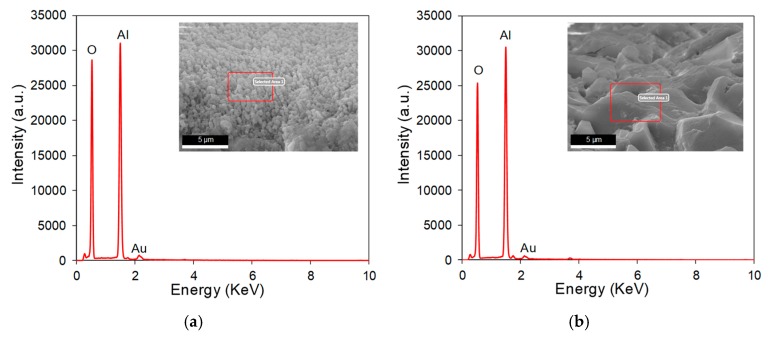
The EDS analysis results for given locations at the entrance sidewall marked by red squares, in which (**a**) represents direct laser trepanning while (**b**) corresponds to SWILT. The involved parameters are identical to those in Figure 4.

**Figure 6:**
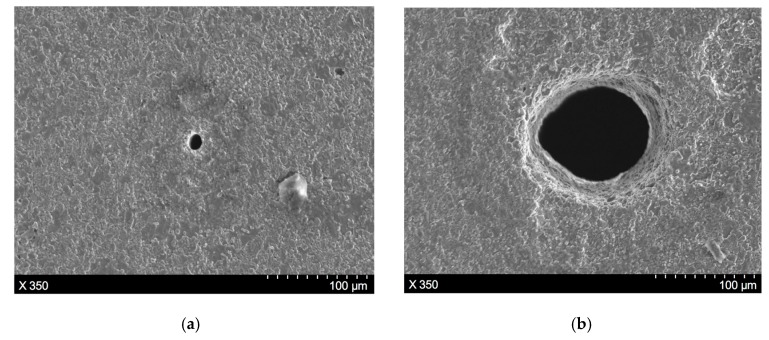
Comparison of micro-through-hole exits, in which (**a**) represents direct laser trepanning and (**b**) corresponds to SWILT. The used parameters are: *f* = 220 kHz, *I* = 2.08 MW/cm^2^, *v* = 200 mm/s, *m* = 300, *n* = 30.

To clearly illustrate the characteristics of the machining results produced by direct laser trepanning and SWILT schemes, a detailed comparison of given ablated surfaces caused by identical processing parameters is made, as shown in Figure 4, where (a) represents the direct laser trepanning and (b) corresponds to SWILT. It can be noticed that the direct laser trepanning result is associated with an obvious hole taper, as the hole entrance is much larger than the exit. In contrast, SWILT results in a nearly straight hole sidewall, and hence a much smaller hole taper, since the diameter difference between hole entrance and hole exit is comparable. In terms of ablated surface quality, dense humps can be observed on the hole sidewall caused by direct laser trepanning, and most of the humps are of size around 100 nm and distributed evenly and regularly. In the semi-water-assisted case, the sidewall surface is much rougher, and irregular morphology features with size scales of several microns can be observed. Figure 5 shows the energy dispersive spectrometer (EDS, OCTANE SUPER-A, EDAX AMETEK, Mahwah, NJ, USA) analysis results at given sidewall positions. It seems that the intensity of oxygen (O) is slightly less in the semi-water-assisted trepanning results compared with the direct trepanning case, which may be attributable to the effective removal of the molten and oxide layers that are induced by laser irradiation via laser-caused mechanical effect in water, such as strengthened shockwaves and high-pressure waterjets originating from water-confined plasma expansion and dynamic bubble collapse [25,26,27,28]. Figure 6 depicts the exit details of the trepanning results, where it can be clearly seen that direct laser trepanning leads to a quite small exit, and therefore a large hole taper is expected, while SWILT is associated with a wider exit and hence a quite straight sidewall, although the roundness is not as good as in the direct trepanning case. Again, this can be attributed to the difference in material-removal mechanisms, where the semi-water-immersed method is associated with obvious mechanical effects caused by the water-confined plasma zone and following bubble cavitation and collapse. Such mechanical effects appear to be more significant at positions approaching the through-hole exit side due to a reduction in space, and leading to reduced hole tapering In contrast, direct laser trepanning causes material removal in a purely thermal way, and the energy loss and material-removal difficulty increase with an increase in hole depth, resulting in obvious hole tapering.

**Figure 7:**
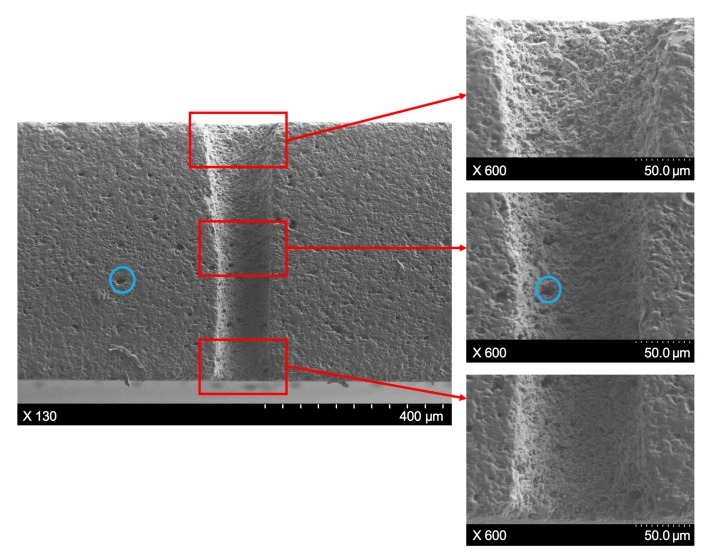
The section plane details of a hole produced by SWILT, where the processing parameters are: *f* = 220 kHz, *I* = 2.08 MW/cm^2^, *v* = 200 mm/s, *m* = 300, *n* = 30.

To further illustrate the hole profile variance along the workpiece depth direction, as well as other machining characteristics of the through-hole, the SWILT result was ground and polished carefully to obtain a section plane containing the hole axis, as shown in Figure 7, which corresponds to same processing parameters as the results depicted in Figure 6b. It is clearly demonstrated that the through-hole possesses a small hole taper, as the hole diameter peaks at the hole entrance and then changes slightly downwards. In addition, the sidewall details at the hole entrance, middle part and the exit position can be observed in Figure 7, where it seems the surface quality of the sidewall does not change with the location, and constant rough surface details can be noted. Meanwhile, there exist many tiny holes or porosities both on the section plane and the sidewall surface (circled in blue), which are formed during the hot-pressed sintering process for alumina ceramic plates from powders. Over the entire section plane, no crack is observed.

### 3.2. Effects of Parameters on Direct and Semi-Water-Immersed Laser Trepanning

After the trepanning experiment designed using the orthogonal scheme, the micro-through-holes were measured, and the averaged diameter readings of exit and entrance, as well as the hole tapering, are listed in Table 4 and Table 5, representing direct and semi-water-assisted laser trepanning results, respectively. In these tables, σ-Entrance refers to the standard deviations of diameters at the entrance, while σ-Exit corresponds to the exit. It can be noted that the SWILT results are associated with larger exits and entrance diameters compared with the direct laser trepanning results, indicating that the former is more efficient in material removal, especially in the lower part of the micro-through-holes. However, the hole produced by SWILT has a worse roundness, since the associated σ-Entrance and σ-Exit are larger than the direct trepanning results. In addition, it should be noted that SWILT is capable of producing a nearly straight hole sidewall, as the hole taper can be reduced to less than 0.2°, which is much less than the direct laser machining level.

**Figure 8:**
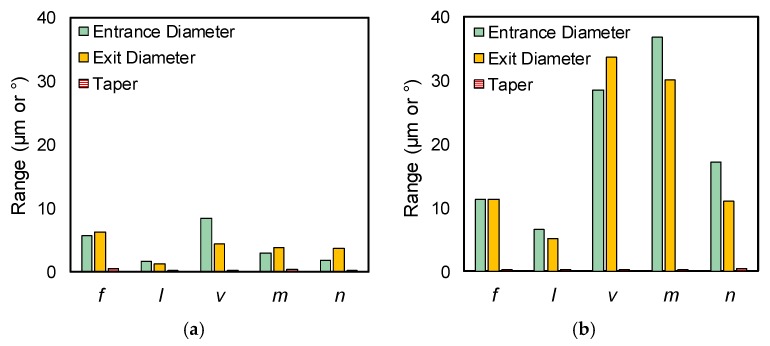
Range analysis results of selected parameters on (**a**) direct laser trepanning and (**b**) SWILT results.

Based on the measured results listed in Table 4 and Table 5, range analysis was conducted (calculation process details can be found in a previous work [16]). Figure 8a,b shows the range analysis results of the chosen processing parameters on the direct laser trepanning and SWILT results (i.e., the entrance/exit diameters and the hole taper). It can be noticed that the range values of SWILT are obviously larger than that of the direct trepanning case. More precisely, for direct laser trepanning, the scanning velocity and laser pulse frequency have relatively significant influences on the entrance and exit diameters, followed by element number, layer number and laser power intensity, while the pulse frequency is associated with noticeable influence on hole tapering while other factors are comparatively insignificant. For the SWILT case, scanning velocity and element number far surpass the others in affecting significance on entrance and exit diameters, followed by layer number, pulse frequency and laser power intensity. Considering the hole tapering, the effects of laser power intensity, element number and layer number are noticeable, whereas the rest have a considerably marginal effect.

**Figure 9:**
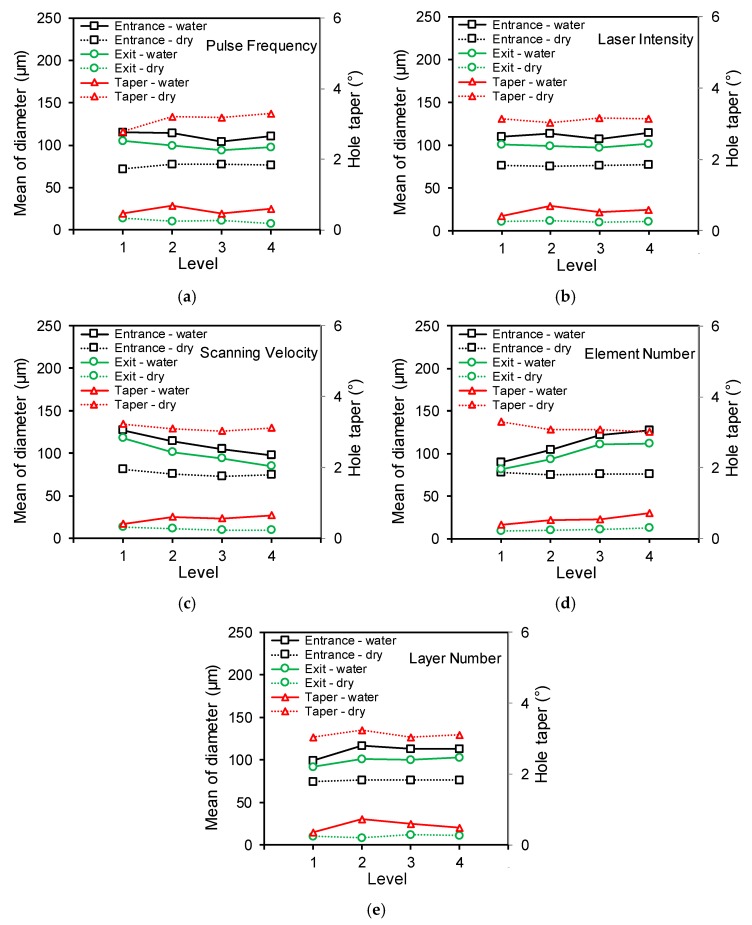
Effects of processing parameters on the trepanning results. (**a**) Pulse frequency, (**b**) laser power intensity, (**c**) scanning velocity, (**d**) element number and (**e**) layer number.

The orthogonal design scheme was employed in this experimental study, and the orthogonal array was properly selected from the well-designed array list. Each factor’s level varies evenly within the given parameter range, and therefore the mean of measured data can reflect the influence of a certain parameter on the trepanning result. Figure 9 shows the changing trends of the measured results (i.e., entrance/exit diameter and hole tapering), with respect to the selected processing parameters, where the dashed line and solid line represent the direct trepanning and SWILT results, respectively. In addition, black, green and red colors are employed to mark trending lines corresponding to entrance, exit and hole tapering, to make them easier to distinguish. Generally, the SWILT scheme results in larger hole entrances and exits and a much-reduced hole tapering compared with the direct trepanning method. This may be attributed to the increased material-removal efficiency caused by the water-enhanced laser-induced mechanical effects inside the limited hole space, leading to more material removal, especially in the area near the exit side (and thus a significantly decreased hole taper).

To ensure the repeatable formation of through-holes rather than blind ones on the alumina ceramic plate—which is difficult to drill through due to the low light absorption coefficient, small laser irradiation area and considerable plate thickness—the processing parameters’ levels have to be properly selected in the available range to guarantee enough repeated laser irradiation times and sufficient input laser energy under different conditions. This may lead to a narrow parameter window, and theoretically result in surplus energy deposition into the machining location. As a result, the trepanning result indicators may not be sensitive to the variance of certain parameters, such as the effects of laser pulse frequency and laser power intensity on the SWILT results, as shown in Figure 9a,b. It is noted in Figure 9c that an increase in laser scanning velocity is associated with decreases in hole exit and entrance diameters for the SWILT results, as well as a slight increase in hole tapering, and similar trends exist for the direct machining results. Both of these may be attributable to reduced energy deposition at certain positions where the laser spot scans over them more quickly, causing less material to be removed in a thermal or mechanical way. Figure 9d illustrates that an increase in element number increases the hole exit and entrance diameters simultaneously, and a slightly increased hole taper is also produced in the SWILT case. In contrast, for the direct machining case, the entrance and exit diameters change marginally with an increase in the element layer, resulting in a slight decrease in hole tapering. Figure 9e shows the changing trend of trepanning indicators with respect to layer number (i.e., the feeding times of the laser focal plane position in workpiece depth direction), and the turning points are observed in the SWILT results. The hole entrance and exit and hole tapering peak at Level 2, corresponding to layer number of 20, which means that when the focal plane position feeds downwards regularly 20 times during the trepanning process, the micro-through-hole exit and entrance, as well as the hole tapering, achieve their maximum values.

Based on the discussion of the processing parameters’ effects on the machining results, optimized parameter combinations can be reasonably derived for SWILT. Specifically, higher laser power intensity and small pulse frequencies are preferred from a point view of increasing the pulse energy so that the alumina ceramics can be drilled through more efficiently and repeatably under different conditions, effectively avoiding possible blind hole formation during the complicated laser-solid-plasma interactions inside a limited space. In addition, a large scanning velocity is necessary to reduce the through-hole diameter and increase the aspect ratio, as less energy is to be deposited into a given position on the material surface when the laser spot sweeps faster over it, leading to less material removal. Similarly, a small element number and layer number should be chosen to reduce the total trepanning time, and thus the accumulated input energy, resulting in smaller hole diameters and, as a result, a higher aspect ratio. Therefore, the optimized parameters are *f* = 200 kHz, *I* = 2.58 MW/cm^2^, *v* = 400 mm/s, *m* = 100, *n* = 15, corresponding to a single-pulse energy of around 110.5 μJ and a theoretical laser peak power intensity at about 1077 GW/cm^2^ within a single-pulse duration, which is high enough to trigger laser-induced breakdown in water.

## 4. Discussion

### 4.1. Potential for Increasing the Aspect Ratio

**Figure 10:**
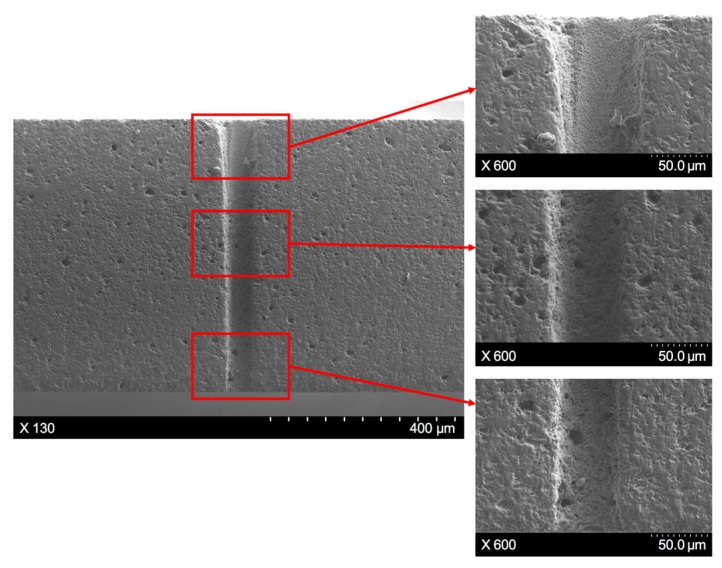
The section plane details of the hole produced using the semi-water-immersed method, where the processing parameters are: *f* = 200 kHz, *I* = 2.58 MW/cm^2^, *v* = 400 mm/s, *m* = 100, *n* = 15.

Since the hole taper produced by the SWILT method is generally small, it is worth investigating its potential for aspect ratio improvement. The section plane, shown in Figure 7, corresponds to an aspect ratio of around 4, where the averaged hole diameter is 155.9 μm, which is estimated by measuring 10 evenly distributed locations over the entire depth direction. Employing the recommended optimized parameter combination (i.e., *f* = 200 kHz, *I* = 2.58 MW/cm^2^, *v* = 400 mm/s, *m* = 100, *n* = 15), a larger aspect ratio with a smaller tapering can be obtained, and the trepanning result is demonstrated in Figure 10. It can be clearly observed that the sidewall is almost straight and the diameter distribution is generally even, resulting in an aspect ratio of nearly 8 (the averaged hole diameter is 79.2 μm). In addition, the surface quality of the sidewall near the hole entrance is better than the corresponding part shown in Figure 7, as a densely distributed, sub-micron-size hump can be noticed, rather than larger particles of several microns, suggesting that the direct laser heating effect may dominate the material removal in this position instead of the mechanical effects. From experimental trepanning tests, it has been confirmed that micro-holes with smaller diameters (and thus large aspect ratios) are harder to drill through, and usually require more trepanning time; therefore, the upper part of the micro-hole may be mainly fabricated by direct laser irradiation before the ceramic plate is penetrated through. In contrast, the sidewall in the middle and lower parts still has a rough surface, indicating that material removal is caused by the mechanical effects associated with the water/sidewall-confined plasma and the following cavitation bubble collapses, since the water can be drawn up to top surface via the micro-hole once the workpiece has been drilled through.

### 4.2. Formation Mechanisms of the Through-Hole

In this section, the formation mechanisms of the micro-through-holes in the direct laser trepanning and SWILT process are discussed according to the surface morphology study of the micro-trepanning results and the investigation into the processing parameters’ influences on through-hole profiles.

**Figure 11:**
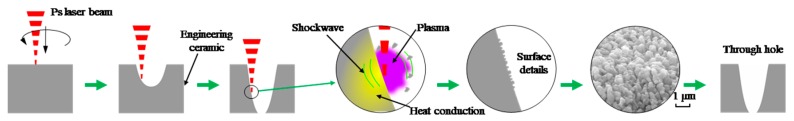
Schematic of the through-hole formation mechanism in the direct laser trepanning of alumina ceramics. The involved SEM figure is captured from Figure 4a.

The through-hole formation process during direct laser trepanning is illustrated in Figure 11, where the material removal is purely attributed to the laser-material interaction, since no assisted media are employed. Depending on the chosen layer number, as the laser beam circles along the set paths at each layer and feeds downwards, the transition blind hole develops gradually, eventually leading to a through-hole if the processing parameters are properly selected, or to a deepened blind hole if the input energy is insufficient or the ablated material cannot be efficiently removed due to a large aspect ratio. Therefore, quite narrow windows for several parameters are used in this study, including high levels of output energy adjusted by HV level, and small levels of laser pulse frequency which significantly affect the single-pulse energy. In addition, the laser scanning path diameter should not be less than 60 μm, otherwise blind holes (instead of through ones) will be produced. In terms of the laser–material interaction, the peak intensity of the ps laser is up to around 1077 GW/cm^2^, which is sufficient to cause phase transition and material ionization. This leads to a plasma zone that is in a high-pressure and high-temperature state, which may enhance the removal of the molten material. The material removal can therefore be attributed to evaporation, melt expulsion and phase explosion [16,24,29]. During pulse intervals, the residual liquid material on the ablated surface re-solidifies, leading to sub-micron humps on the sidewall surface. It should be noted that the hole tapering is obvious, which should be attributable to increases in laser energy loss and removal difficulty of the ablated material with incremental hole depth.

**Figure 12:**
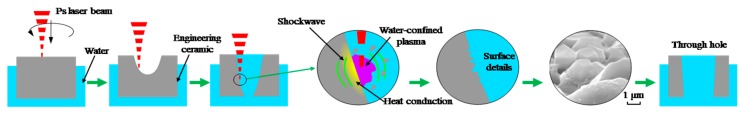
Schematic of the through-hole formation mechanism in the semi-water-assisted laser trepanning of alumina ceramics. The involved SEM figure is captured from Figure 4b.

For the SWILT conditions, the engineering ceramic plate is semi-immersed in water, which has no effect on the trepanning process until the plate is drilled through. Via the drilled through-hole, the surrounding water can be drawn up due to a siphoning effect, and laser-induced breakdown in water may occur. This results in a water-confined plasma zone and subsequent cavitation bubbles, leading to a strengthened mechanical effect and high-pressure micro-sized waterjet, where the pressure can be up to around several GPa. This is sufficient to cause the removal of the softened ceramic particles/powder [25,26,27,28], so that the rough ablated surface may be produced, as illustrated in Figure 12. Since the mechanical effect is stronger in tighter spaces, the material-removal rate near the exit side surpasses that of the upper position, so that a much straighter sidewall is expected, and thus a reduced hole tapering.

**Figure 13:**
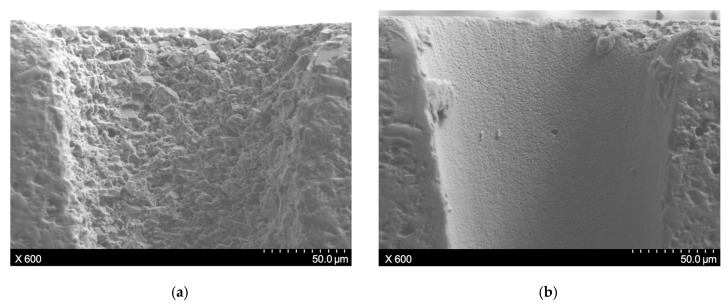
(**a**) The surface details of the hole entrance sidewall machined by SWILT, where the processing parameters are: *f* = 220 kHz, *I* = 2.08 MW/cm^2^, *v* = 200 mm/s, *m* = 300, *n* = 30. (**b**) Same position after post-processing via direct laser trepanning using identical parameters.

To clearly demonstrate the difference in surface characteristics caused by thermally and mechanically induced material removal, the through-hole produced by the SWILT method was post-processed adopting the direct laser trepanning way, and the change of the sidewall details near the hole entrance are illustrated in Figure 13, where the rough surface full of particles (the size of several microns) is transferred into a smooth surface with densely distributed humps (the size of sub-microns), fully explaining the machining features of the two methods.

## 5. Conclusions

A SWILT method has been proposed and investigated experimentally on alumina ceramics employing an orthogonal design scheme, and corresponding direct laser trepanning tests were also carried out for a comparison purpose. The micro-trepanning results were assessed quantitatively via diameter measurement, and qualitatively through SEM/EDS observation. In addition, the influences of the processing parameters on the micro-trepanning results were analyzed, and the through-hole formation mechanisms were adequately discussed both for the direct trepanning and SWILT. The main conclusions are listed as:

- Direct laser trepanning is associated with large hole tapering, with entrance diameters between 70 and 85 μm, while the exit diameter is always less than 20 μm, leading to tapers around 2.6°–3.5°, although the sidewall surface is of good quality and coated with densely distributed sub-micron humps. In contrast, SWILT is capable of producing through-holes with much smaller tapering—down to about 0.19°—and higher material-removal efficiency, while rougher ablated surface full of particles (the size of several microns) can be noted. By using the optimized processing parameters, a straight through-hole with averaged diameters around 79.2 μm and an aspect ratio about 8 can be repeatably produced.
- Effects of processing parameters on the trepanning results have been assessed through range analysis, where the through-hole profile changes were more significant in SWILT than the direct trepanning case under same conditions. In terms of direct laser drilling, laser scanning velocity and pulse frequency have obvious effects on exit and entrance diameters, whereas hole tapering is mostly affected by pulse frequency. For SWILT, the laser scanning velocity and element number far surpass other parameters in affecting the exit and entrance diameters, while the hole tapering is constantly kept at a quite low level. Recommended parameters have been made based on the influence analysis for SWILT, including a smaller pulse frequency (Level 1), higher laser power intensity (Level 4), larger laser scanning velocity (Level 4) and fewer element numbers and layer numbers (Level 1).
- The micro-hole formation mechanisms in the two trepanning methods have been adequately analyzed based on experimental observation. For the direct laser trepanning case, the transition blind hole develops as the incident laser beam interacts with material and causes material removal in a purely thermal-dynamic manner, leading to a deepening transition hole until a through one is eventually formed. The densely distributed sub-micron hump on the sidewall surface may be attributed to the re-solidification of the residual molten material after laser pulse heating. In contrast, for SWILT, once the alumina ceramic plate is drilled through, water can be drawn up due to a siphoning effect and fill the hole, so that a laser-induced breakdown in water is occurs. This is followed by water-confined plasma and associated strengthened mechanical effects, which may cause the material removal of molten or softened layers, leading to an increased material-removal rate especially in tighter spaces (and hence a straighter sidewall). Meanwhile, a degenerated surface quality of the sidewall is also expected.

In this study, the SWILT method has shown good potential in producing micro-through-holes on alumina ceramics, and is able to yield large aspect ratios, minimized hole tapering and high machining efficiency. The surface quality of the ablated sidewall can be improved through post-processing methods such as direct laser irradiation, as indicated in this study, and will be further explored in future investigations.

## Figures and Tables

**Table 1:** Material properties of 95% alumina ceramics [14].

Properties	95%
Density (kg/m^3^)	3720
Melting point (K)	2323
Vaporization point (K)	3253
Elastic module (GPa)	300
Tensile strength (MPa)	220
Compressive strength (MPa)	2600
Specific heat (J/kg K)	770
Thermal conductivity (W/mK)	38
Coefficient of thermal expansion (10^−6^/K)	8.2

**Table 2:** Main components in 95% alumina ceramic plate.

Components	Ratio
Al_2_O_3_	94–95%
SiO_2_	2%
CaO	2%
MgO	1%

**Table 3:** The processing parameters selected in this study.

Machining Parameters	Abbreviations	Levels			
		1	2	3	4
Pulse frequency (kHz)	*f*	200	220	240	260
Laser power intensity (MW/cm^2^)	*I*	2.08	2.34	2.47	2.58
Scanning velocity (mm/s)	*v*	100	200	300	400
Element number	*m*	100	200	300	400
Layer number	*n*	15	20	25	30

**Table 4:** Summary of experiment results in direct laser trepanning.

Test No	*f*	*P*	*v*	*m*	*n*	Entrance Diameter (µm)	𝜎-Entrance (µm)	Exit Diameter (µm)	𝜎-Exit (µm)	Taper (°)
L1	1	1	1	1	1	77.72	3.62	14.48	4.95	3.02
L2	1	2	2	2	2	70.27	4.70	12.03	1.23	2.78
L3	1	3	3	3	3	69.18	5.53	13.70	1.54	2.65
L4	1	4	4	4	4	71.32	5.42	14.87	1.58	2.69
L5	2	1	2	3	4	77.23	4.25	11.55	1.14	3.13
L6	2	2	1	4	3	81.83	3.99	17.42	3.61	3.07
L7	2	3	4	1	2	78.94	10.70	4.39	6.23	3.55
L8	2	4	3	2	1	72.87	3.57	8.75	6.34	3.06
L9	3	1	3	4	2	74.82	2.82	8.80	1.56	3.15
L10	3	2	4	3	1	73.67	1.49	10.53	2.04	3.01
L11	3	3	1	2	4	82.61	3.67	13.32	2.83	3.30
L12	3	4	2	1	3	79.85	2.72	10.71	1.80	3.30
L13	4	1	4	2	3	73.80	3.35	6.46	1.28	3.21
L14	4	2	3	1	4	74.39	2.70	5.58	4.14	3.28
L15	4	3	2	4	1	75.22	3.47	9.42	1.02	3.14
L16	4	4	1	3	2	82.63	3.17	8.53	1.67	3.53

**Table 5:** Summary of experiment results in SWILT.

Test No	*f*	*P*	*v*	*m*	*n*	Entrance Diameter (µm)	𝜎-Entrance (µm)	Exit Diameter (µm)	𝜎-Exit (µm)	Taper (°)
L1	1	1	1	1	1	97.41	4.42	100.99	7.37	−0.17
L2	1	2	2	2	2	120.38	19.49	102.89	10.35	0.83
L3	1	3	3	3	3	119.45	12.71	109.13	8.84	0.49
L4	1	4	4	4	4	123.86	11.08	109.16	9.06	0.70
L5	2	1	2	3	4	129.43	7.28	118.35	7.73	0.53
L6	2	2	1	4	3	150.45	11.78	131.50	8.55	0.90
L7	2	3	4	1	2	82.78	6.87	66.64	7.35	0.77
L8	2	4	3	2	1	93.91	8.79	83.22	8.42	0.51
L9	3	1	3	4	2	118.69	12.41	104.25	8.27	0.69
L10	3	2	4	3	1	93.04	7.29	82.28	7.73	0.51
L11	3	3	1	2	4	112.12	5.18	108.19	5.43	0.19
L12	3	4	2	1	3	91.78	6.45	82.22	7.41	0.46
L13	4	1	4	2	3	92.00	10.26	79.98	7.55	0.57
L14	4	2	3	1	4	88.35	6.42	77.25	8.18	0.53
L15	4	3	2	4	1	114.88	8.26	102.36	10.59	0.60
L16	4	4	1	3	2	146.01	9.56	132.07	7.31	0.67
